# Detection of small microplastics in the surface freshwater samples of Yangcheng Lake, China

**DOI:** 10.1016/j.heliyon.2024.e39779

**Published:** 2024-10-28

**Authors:** Zhenyu Xu, Natalie Earnhardt, Domna G. Kotsifaki

**Affiliations:** Photonics Lab, Division of Natural and Applied Sciences, Duke Kunshan University, 8 Duke Ave, Kunshan, 215316, Jiangsu Province, China

**Keywords:** Sub-20 μm plastics, Crab-culturing lake, Optical tweezers confocal micro-Raman spectroscopy, Aqua environment, Plastic polymers

## Abstract

Microplastics up to 20 μm are recognized as having the highest potential to cause significant impacts on aquatic environments. Current methods face challenges in detecting and chemically characterizing small microplastics in freshwater systems. In this study, using an optical confocal micro-Raman tweezer technique, the composition of particles trapped in lake aggregates collected from surface water around Yangcheng Lake in Suzhou, China, has been identified. Surface freshwater samples were analyzed from 15 different sites around the lake. In total, 514 particles were analyzed of which 136 were small microplastics. Chemical characterization showed the presence of five different polymer types, with polystyrene being the most dominant, accounting for 63% of the detected particles. Small plastics in the range of 1.1 to 8.5 μm were detected around crab restaurants and residential villages. The smallest microplastics identified were 1.1 μm polystyrene. Fragment was the most common shape of microplastics, followed by fiber and quasisphere within the volume of sample analyzed. The results suggest that the primary sources of small microplastic contamination in Yangcheng Lake may include fishing activities, agriculture, and tourism. Study findings may be used as a reference to extend the understanding of the small microplastic contamination level in inland freshwater systems.

## Highlights

• Small microplastics have been detected in the Yangcheng Lake, China.
• A method based on Raman spectroscopy and optical tweezers has been used.
• Five types of polymers (PS, PE, PVC, NY6, PMMA) have been detected.
• Fragments are the dominant shape.
• Microplastic contamination in Yangcheng Lake may be caused by human activities.

## Graphical abstract

**Figure:**
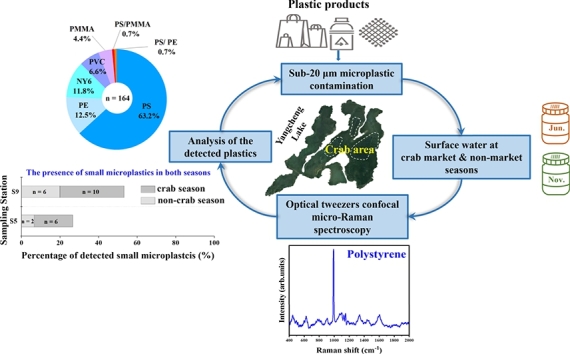

## 1 Introduction

Nowadays, plastic polymers are mass-produced due to their various desirable properties, such as durability, lightweight nature, and relatively low cost of production [1]. They have brought convenience to human life but their widespread use has raised significant ecological and environmental concerns. It has been reported that the total amount of plastic produced globally was around 391 million metric tons (Mt) in 2021 [2] and it is estimated that 12,000 Mt of plastic waste will be released into the environment by the end of 2050 [3]. There, under various processes of degradation such as photodegradation, oxidation, hydrolysis, and mechanical crushing, large plastics can break down into smaller fragments *i.e.* microplastics (less than 5 mm), sub-20 μm plastics (20 μm - 1 μm) [4], [5] and in some cases in nanoplastics (less than 1 μm) [6], [7]. Due to their small size, they can be easily transported from land to the ocean where these plastics often travel long distances and persist for a long time. In addition, they can enter into trophic chains through bio-accumulation. Recently, their occurrence has been reported in drinking water [8], soil [9], zooplankton [10], the gastrointestinal tract of consumed fish species [11], human placenta [12] and human peripherical blood [13]. Therefore, a comprehensive understanding of the distribution of small plastics in the environment may allow us to develop effective strategies to protect both ecosystems and human health.

Since small plastics are present in a variety of environmental matrices, their successful characterization requires a careful selection of methodologies for sample pretreatment, detection, identification, and quantification. So far, well-assessed protocols and detection methods have been developed for qualitative and quantitative analyses of microplastics. These include Raman spectroscopy [14], [15], Fourier-transform infrared (FTIR) spectroscopy [16], [17], scanning electron microscopy (SEM) [18], [19], dynamic light scattering [20] and so on. For example, Raman spectroscopy identifies microplastics using the characteristic vibrations of molecules as a detectable feature and obtaining detailed chemical information about target samples. Meanwhile, the spatial resolution of conventional Raman spectroscopy is hindered by the diffraction limit, resulting in the identification of microplastics a few μm in size. On the other hand, FTIR spectroscopy is a non-destructive technique for measuring plastics of irregular shapes and sizes. However, due to absorption losses by water molecules in the infrared regime, plastics characterization in liquids is limited. Thus, it is necessary to utilize a more precise identification approach that can detect small plastics embedded in organic matter in aquatic environments. Optical tweezers use the forces that light exerts to manipulate small particles from 10 nm to 30 μm in diameter [21]. They can isolate and study individual particles and cells without physical contact, minimizing the risk of contamination, and simultaneously enabling the analysis at the single-particle level. They have been used in many research areas ranging from physics [22], [23] to biology [24], [25]. Recently, the integration of optical tweezers with micro-Raman spectroscopy allowed for the detection of sub-20 μm plastics around Okinawa, Japan [5]. The authors employed optical tweezers to trap single microplastic particles in liquid environment and used Raman spectroscopy to obtain their chemical spectra. This approach allowed for the identification of microplastics in ocean water, without the need for extensive sample preparation. By eliminating the need for additional procedures to remove organic matter, this method not only simplifies the analysis process but also reduces both chemical and material waste.

Plastic pollution in China has attracted considerable attention with extensive investigations to be conducted in sea [26], [27], [28], [29], rivers [30], [31] and lakes [32], [33], [34], [35]. Lakes play an important role in inland ecosystems and biogeochemical cycles and have been subjected to industrialization, urbanization, and agricultural industrialization [36], [37]. As such, lakes may serve as a major sink for plastics in freshwater ecosystems, since plastic debris can accumulate and persist over a long period of time [36], [37]. Nowadays, studies have examined the microplastic contamination in many freshwater ecosystems, such as Mongolia Lake [38], Taihu Lake [39], Poyang Lake [40], Dongting Lake [41], Hong Lake [41], China, and worldwide [36]. These studies have predominantly found plastic particles ranging in size from 20 μm to less than 2 mm [36], [37]. For instance, microplastics detected in Taihu Lake [39] were mostly fibrous, with a size of 100 μm - 1000 μm and mainly composed of cellophane, while in Poyang Lake [40] most were polypropylene (PP) and polyethylene (PE) with a size of 50 μm - 5000 μm, and fiber in shape. In addition, in Kallavesi Lake [42], Finland, the microplastics were found to be between 20 μm and 300 μm, while in Yenagoa Lake [43], Nigeria, microplastics detected during the rainy season ranged from 0.51 mm to 1.00 mm. Note that several factors can affect the sizes of microplastics, including the degree of degradation, exposure to environmental conditions (*i.e.*, UV radiation, temperature), as well as the physical and chemical properties of the plastic material [44]. Furthermore, the methodology used for sample collection and analysis [36], [37], including the mesh size of filters and detection techniques, also plays a significant role in determining the reported size distribution of microplastics. In addition, small microplastics tend to have a large specific surface area, allowing micro-organisms to attach and form biofilms. Hence, small plastics can be easily translocated within organisms and exhibit high toxicity [44]. Consequently, investigating the presence of small-sized microplastics in the environment is essential for accurately assessing ecological risks.

Yangcheng Lake in Suzhou city is a medium-sized lake with an average depth of around 2.5 m and a water area of 120 $Km^{2}$. The characteristic of being an open basin with many rivers flowing in and out enables the transfer of microplastics between different water areas making Yangcheng Lake an ideal place to study the small-sized microplastic contamination. In this work, the microplastic contamination in Yangcheng Lake using optical tweezer confocal micro-Raman spectroscopy (OT*μ*-RS) was studied to reveal the occurrence, polymer type, average size, and shape of small microplastic particles. The microplastic contamination was investigated during the crab market season (crab season) and non-market season (non-crab season). In the chemical composition analysis, polystyrene (PS) and polyethylene (PE) were the major polymer types of the selected plastic particles, indicating that human activities such as fishing, as well as the topographic factors might be the sources of small plastics in the lake. The majority of the evaluated microplastics were found with a size in the range of 1.1 - 8.5 μm for the quasisphere plastics, with the smallest microplastic being 1.1 μm polystyrene. The microplastics were divided into three shapes *i.e.*, quasisphere, fragment, and fiber, of which fragment was the most abundant shape of the evaluated microplastics. The purpose of this study is to use an alternative detection method to determine the extent of sub-20 μm microplastics contamination in an area with significant anthropogenic activities so that it may assist in extending our knowledge regarding small microplastics contamination in inland freshwater systems.

## 2 Materials and methods

### 2.1 Study area and sample collection

**Figure 1:**
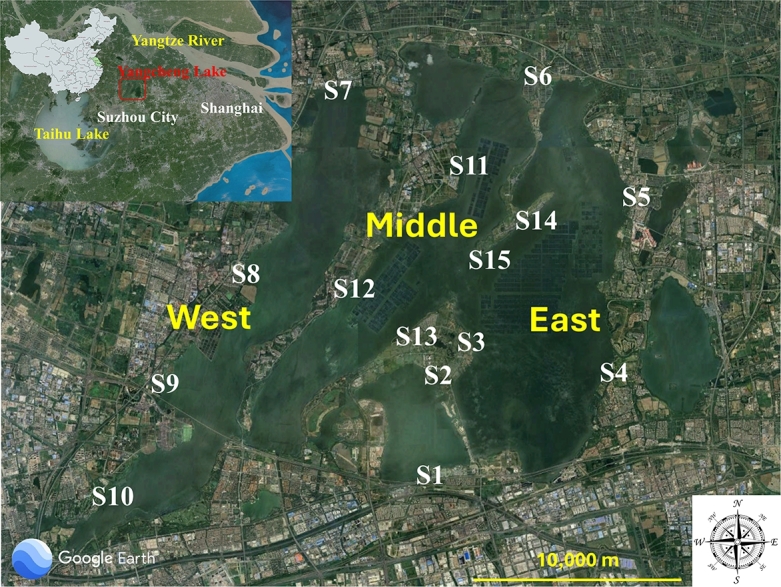
Map of Yangcheng Lake and sampling sites from which particles were collected and analyzed using optical tweezers confocal *μ*-Raman spectroscopy. Fifteen sampling points around the Yangcheng Lake were chosen to map the plastic distribution. The shadow square areas distributed inside the lakes are crab-culturing areas (S11, S12, S14, and S15). Inset: Map of the People's Republic of China in which the green area shows the Jiangsu Province. The geographical location of Yangcheng Lake is noted with the red rectangle [46].

Yangcheng Lake (center at N31°43', E120°17') is a water source located between Taihu Lake and the Yantze River, in the northeast region of Suzhou, Jiangsu Province, China (Fig. 1). The morphology of the lake body is relatively special and divided into three zones by two belt-shaped sand ridges [45], including west (32.0 $Km^{2}$), middle (34.6 $Km^{2}$), and east (52.5 $Km^{2}$) with a total area of about 120 $Km^{2}$. In addition, out of the three zones, rivers enter into the west zone and out of the east zone. Since the lake is located in the southern part of Jiangsu Province, it exhibits a humid subtropical climate. Yangcheng Lake serves as the primary water source for Suzhou and Kunshan [45], and its aquaculture industry has experienced substantial development. Furthermore, Yangcheng Lake is the most famous Chinese hairy crab-producing area in China. The Chinese hairy crabs culture areas are mainly located in the northeast and southwest of the middle zone (S11 and S12), and in the east zone (S14 and S15) as shown in Fig. 1. Therefore, with the economic development of the areas around the lake, a large number of land-based pollutants may be discharged into the freshwater.

Fifteen sampling sites were chosen to provide a road map of the plastic contamination distribution of Yangcheng Lake (Supporting Information S1-Table S1 and Fig. 1). The freshwater samples were collected over 24 h in June 2023 and November 2023. All of the samples were collected using Kemmerer bottles at a depth of 50 cm below the water surface, stored in 237 mL glass bottles and sent to the laboratory for analysis. Note that cotton clothes and rubber gloves were worn during the collection of environmental water samples. All samples were stored in a climatized environment until processing the next day.

### 2.2 Laboratory analysis

Each of the stored freshwater samples was filtered with a 125 μm stainless-steel sieve to remove large microplastics as well as macroplastics, and the remaining water solutions were stored in new, clean glass bottles. No additional chemical steps were added to remove the organic matter. A small volume of liquid from each of these remaining freshwater solutions was collected and examined using an optical microscope. Small microplastics were observed in all 15 sample sites. Note that during sampling, sample preparation, and analysis direct contact with the freshwater solution samples was avoided. All glass bottles were ultrasonically washed for 30 min in Milli-Q water and repeatedly rinsed with Milli-Q water before use. To ensure that there was no additional plastic contamination during sample processing and analysis, Milli-Q water was also used as a blank sample. A microwell was created on the microscope glass slide using adhesive microscope spacers, and a 10 μL sample solution was placed on it and sealed with a glass cover slide. The microscope slide was mounted and fixed on the top of the translation stage of the optical tweezers confocal *μ*-Raman spectroscopy system.

### 2.3 Optical tweezers confocal *μ*-Raman spectroscopy system

The optical tweezers confocal *μ*-Raman system (LabRAM HR Evolution HORIBA Scientific) consists of a diode laser beam at 532 nm with a maximum power of 54 mW to provide better Raman efficiency compared to longer wavelengths. The laser beam was focused using a high numerical aperture (NA = 1.25) oil immersion objective lens (Plan N 100× Olympus) onto the sample. The high NA of the lens provided both the necessary laser intensity needed to maximize its Raman signal and generate a strong gradient force.

Optical tweezers use a highly focused laser beam to manipulate particles by balancing two forces: the gradient force, which pulls particles toward the focal point of highest light intensity, and the scattering force, which pushes them along the direction of light propagation [21]. Stable trapping requires a gradient force greater than a scattering force [21]. In order to achieve this, an objective lens with a high numerical aperture is required to create a steep intensity gradient, leading to a large gradient force [21]. It is also necessary for the particle to have a refractive index greater than its surroundings [21] (a condition usually met by plastic polymers in water solutions [47]). Furthermore, microplastics can be influenced by thermal effects induced by the focused laser beam, causing microplastic particles to move toward the laser beam and be trapped under the influence of the optical forces [21]. This movement may assist in capturing and positioning the particles within the beam's focal point. Therefore, for the chemical analysis of microplastics in liquid environments, stable trapping is essential, as it ensures the particles remain immobile within the trapping point and are not picked up from the freshwater solution, allowing the Raman signal to be collected [48].

Spectra were collected in the range of 400 - 2000 $cm^{-1}$ using an acquisition time of 15 s. A CCD camera was used to collect images of the trapped plastic during the experimental process. Furthermore, when the laser beam is blocked the trapped particle is released from the trapping point in the freshwater sample and imaged again. By re-illuminating a new particle is trapped in the same sample solution and a new image is collected. The images were evaluated using ImageJ software to determine the size and form of the microplastics and to ensure that the study was carried out on a new plastic particle in the same sample solution. Raman spectra were collected from the system and analyzed. The analysis included baseline correction before peak position determination. Then, the Savitzky–Golay filter was used for spectra smoothing by a 3rd-degree polynomial function.

## 3 Results and discussion

### 3.1 Chemical composition of sub-20 μm microplastics

**Figure 2:**
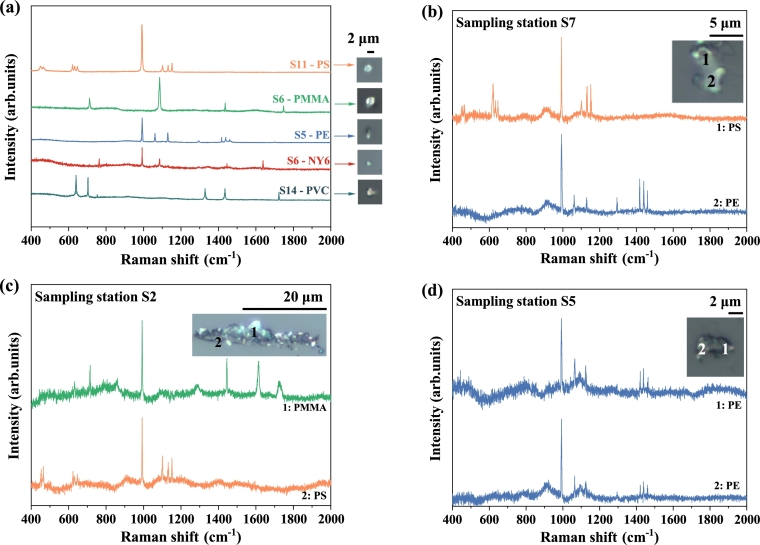
(a) Raman spectra of sub-20 μm microplastic particles detected in Yangcheng Lake with their optical images. (b) Raman spectra of a sub-20 μm microplastic fragment of 6.9 μm long side size and 4.6 μm short size, found at station S7, *i.e.* Waihetou village, whose part noted as (1) polystyrene (PS) and part noted as (2) polyethylene (PE). (c) Raman spectra of a microplastic fragment of 25.4 μm long side size and 4.9 μm short side size, found at station S2, *i.e.* Chongyuan temple, whose part noted as (1) polymethyl methacrylate (PMMA) and dark part noted as (2) polystyrene (PS). (d) Raman spectra of polyethylene (PE) microplastic fragment plastic of 4.6 μm long side size and 2.1 μm short side size, found at sampling S5, *i.e.* crab restaurants.

Raman spectra of each type of polymer that was chemically identified from the Yangcheng Lake freshwater samples are shown in Fig. 2. Five polymer types of sub-20 μm plastics were detected in the freshwater, which were polystyrene (PS), polyethylene (PE) polyvinyl chloride (PVC), polyamide-6 (NY6) and polymethyl methacrylate (PMMA) (Table 1, Fig. 2 (a), and SI-S2). In Table 1 the hazard score of each plastic [49] is provided to indicate their potential health and environmental impact [49]. Figs. 2 (b) and (c) show the Raman spectrum of a single microplastic particle composed of different polymers at different positions. Fig. 2 (d) shows the Raman spectrum of a PE microplastic fragment of 4.6 μm long side size and 2.1 μm short side size collected at different locations on plastics found at station S5. The variation in intensity shows how the material is weathering differently at different locations [5]. At the same time, some Raman spectra of a microplastic particle shown large fluctuations and additional peaks which could be organic matter [5] (SI-S3).

**Table 1:** **Polymer types found as microplastics in Yangcheng Lake freshwater samples for both seasons.** Detailed information for polymers characterized in this study, including hazard score [49], sample sites where the small microplastics are detected, and the number of small microplastics for each polymer type. Note that copolymerized plastics were not included.

Polymer Type	Hazard score	Sampling site	Number of plastics
Polystyrene (PS)	30	S1-S3, S5-S15	86
Polyethylene (PE)	11	S1, S3, S4, S6, S7	17
Polyvinyl chloride (PVC)	10,551	S4, S5, S9, S10, S14	9
Nylon-6 (Polyamide-6) (NY6)	50	S4, S6-S12, S14	16
Polymethyl methacrylate (PMMA)	1021	S2, S3, S6	6

Polystyrene (PS) is found in the surface water of all of the sampling sites at Yangcheng Lake except station S4 (Fig. 1 and Table 1). PS is widely utilized in the manufacturing of food containers, including trays, plates, cups, *etc.* [50], [51]. One possible reason for finding many small PS microplastics within the sample volume could be due to anthropogenic activities, particularly the existence of restaurants in the vicinity, suggesting a potential correlation with the food packaging practices. Polyethylene (PE), was detected in five sampling sites (Table 1). It is used for the disposal of products like plastic bottles, bags, and bottle caps [41]. This could explain the abundance of PE detected in stations near Huayi Film Center (S1) and Hotel area (S3) (Fig. 1). PE particles were also detected at stations S5 and S9 during the crab season, possibly because of the debris from fishing nets and lines [52]. PE products are typically buoyant and easily carried by water due to their low densities, which may further contribute to the polymers' widespread dissemination [41].

Polyvinyl chloride (PVC) is one of the popular plastics worldwide, used in cables, pipes, and fittings [5]. Although PVC has a larger density (1.38 g/$cm^{3}$) [41] than water, its unique forms and high surface-to-volume ratios enable it to be found in surface waters [41]. Furthermore, a combination of environmental conditions like temperature, wind, and waves may cause this denser plastic to cycle from deeper water to the surface [53], [54]. PVC also breaks up more easily than other thermoplastics [55]. In this study, PVC was detected in five sampling sites (Table 1). It should be noted that PVC was also detected at sampling station S9 in both collection periods (crab and non-crab seasons). Nylon-6 (NY6), also known as polyamide-6, was detected at nine sampling stations in this study (Table 1). NY6 polymer usually comes from fibers shed when washing textiles [56], which might explain its detection at stations S7, S8, S9 and S12 *i.e.* near the villages. Another source of NY6 can be the abandoned fishing gear [57] used at regions where fishing and/or crabbing activities are frequent (S11, S12, and S14).

Polymethyl methacrylate (PMMA) has been detected in zooplankton in the Yellow Sea [58] and fish in the North Sea [59]. In this study, PMMA small microplastics were found at stations S2, S3 and S6. PMMA is a high-density plastic that is frequently used in various products due to its remarkable qualities like hardness, transparency, and aesthetic appeal [60]. Therefore, the source of PMMA microplastics may be caused by the construction, renovation, and maintenance activities in these regions, which produce dust and fragments that can end up in liquid environment.

The results of this study confirmed that a significant variation in polymer types between the fifteen sample stations was observed. The polymer composition in Yangcheng Lake was found to be similar to that of many other lakes in China [36,37], for instance, Taihu Lake [39], urban lakes in Wuhan [61], Dongting Lake [41], lakes in Tibet plateau [35]. This similarity in polymer composition across these freshwater lakes may indicate common sources of microplastic contamination, likely linked to the widespread use of these polymers in modern life.

### 3.2 Occurrence of sub-20 μm microplastics

**Figure 3:**
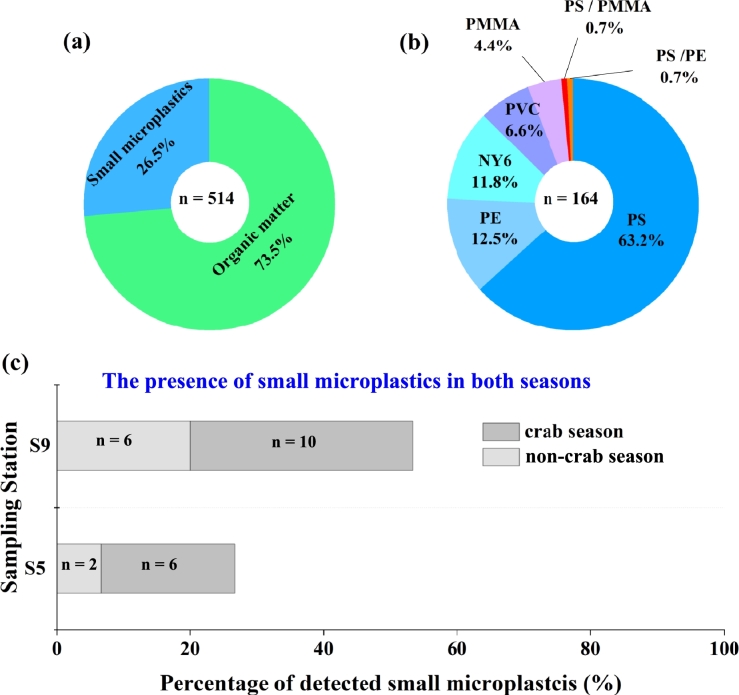
**Microplastic occurrence in surface Yangcheng Lake freshwater samples.** (a) Percentage composition of detected particles. (b) Percentage of polymer type of detected small microplastics collected in freshwater samples of Yangcheng Lake, China. The most dominant plastic is polystyrene (PS) (63.2%). (c) Percentage of the detected small microplastics during non-crab and crab seasons. Include the number of detected plastics.

A total of 514 particles were evaluated, of which 136 (26.5%, Fig. 3(a)) were small microplastics. Polystyrene (PS) is the most dominant plastic discovered, which was identified in almost all sampling sites within the sample volume analyzed (63.2%) (Fig. 3 (b)). Following PS are PE and NY6 which are detected in five and nine sampling sites, accounting for 12.5% and 11.8% respectively, of all detected microplastics. PVC was found in five sampling sites, about 6.6% of all microplastics, while PMMA was detected in three sampling sites, accounting for 4.4% of all detected plastics. Particles that are copolymerized of PS/PE (1 particle) (0.7%) and PS/PMMA (1 particle) (0.7%) were also detected.

Fig. 3 (c) shows the percentage of microplastic occurrence between non-crab and crab seasons at stations S5, *i.e.* crab restaurants, and S9, *i.e.* Zhongxiang village. An increase in the occurrence of small plastics during crab seasons is observed at sampling stations S5 and S9, with an increase of 4 small microplastics per station (SI-S4) in the case of the two selection seasons (June and November). The crab restaurants (S5) are a cluster of restaurants where crab is the main food selling point. During the non-crab market period, crabs are fed and contained within isolation zones made of plastic nets and ropes, therefore we assume that the main source of microplastics is the decomposition of these plastic products in isolation zones in the lake after long-term immersion. During the crab marketing period, the increased concentrations of microplastics (S5 and S9) could be due to increased human activities, including more frequent crab fishing (use of crab pots, boat voyaging), and disposable packaging.

Table 2 shows the occurrence of detected microplastics for each sampling site during both seasons. Small microplastics are most dominant in S14 and S15 (crab-culturing areas) with 16 and 14 plastics, respectively. Following stations S12, *i.e.*, Nan village which is close to the crab farming region, and S7, *i.e.*, Waihetou village, where 11 plastics were chemically characterized in each of these stations. While in stations S2 and S5 (non-crab period), 3 and 2 plastics were identified, respectively. Overall, the occurrence of microplastics varied from 2 to 16 plastics within the sample volume analyzed, with an average occurrence of 8 plastics during non-crab season.

**Table 2:** **The distribution of small micro-sized particles in the freshwater sample from Yangcheng Lake, China (SI-S5)**.

Station Name	Volume checked (μL)	Number of detected microplastics	Number of detected organic particles	Percentage of microplastics (%)
S1	60	9	21	30.00
S2	60	3	28	9.68
S3	60	7	23	23.34
S4	60	6	24	20.00
S5 no crab season	60	2	28	6.67
S5 crab-season	60	6	24	20.00
S6	60	10	20	33.34
S7	60	11	19	36.67
S8	60	8	22	26.67
S9 no crab-season	60	6	24	20.00
S9 crab-season	60	10	20	33.34
S10	60	4	29	12.12
S11	60	7	23	23.34
S12	60	11	19	36.67
S13	60	6	24	20.00
S14	60	16	14	53.34
S15	60	14	16	46.67

Another parameter that may contribute to the microplastic occurrence could be the field farming activity caused by the villages located around the lake (SI-S6). In this study, four village sampling stations (S7, S8, S9, and S12) are located along the river channels branching out of Yangcheng Lake. Additionally, most of the buildings in these villages are old residential houses built along the small water channel. Among these stations, S7 and S12 are most dominant with microplastics (Table 2). The land type layout in S7 is specifically different from the others; the only farmland in station S7 is directly adjacent to water channels and surrounded by a large number of residential houses, while farmlands in other villages are larger in quantity and size, and are also not directly surrounded by residential houses (SI-S6). This might suggest that the field farming activities in S7 are more geographically concentrated than in other villages. Meanwhile, residential houses in stations S7 and S12 are the most densely packed among all villages. These two abovementioned factors, *i.e.*, farming activities and excessive house density close to the water channels, may be the cause of the microplastic occurrence in these areas (S7 and S12).

Plastics detected in stations S1, S3 and S11, and S13 (Fig. 1 and Table 2) might be caused by the use of a large number of disposable products, such as food packaging, toiletries and packaging, sacrificial products, and plastic bags caused by the entertainment and related facilities. It should be noted that station S11 *i.e.*, Hotel which is close to the crab farming area, is under construction and is also close to an industrial area that involves leather and plastic manufacturing, paper companies, lighting appliances, and electronic technology. These activities might explain the small microplastic concentration in station S11 within the freshwater volume analyzed.

Consequently, the occurrence of microplastic particles observed across sampled volumes from different stations highlights distinctions between urban areas, regions with intensive crab farming activities, and those with fewer crab farming activities. Furthermore, the microplastic contamination within the sampled volume of Yangcheng Lake reveals a significant correlation with various anthropogenic activities, particularly those related to the fishing industry and tourism. The lake's geography and hydrodynamics also play a critical role in the distribution of microplastics. Rivers entering the west zone and exiting from the east zone of Yangcheng Lake influence the flow and accumulation of plastics. Urban runoff, industrial discharges, and agricultural activities in the region contribute to the microplastic load entering the lake. Once these microplastics enter the lake, the water flow dynamics help distribute them across different zones. This study highlights that the highest concentration of microplastics within the volume analyzed is found in the middle zone of the lake. This area is particularly noteworthy due to its extensive crab culture activities. Crab farming involves the use of various plastic materials, such as cages, ropes, and feeding equipment. Over time, these plastics degrade into smaller particles, contributing to the small microplastics observed. Additionally, during crab season, plastic material is used more often in crab farming, resulting in an increased amount of small microplastics. Furthermore, Yangcheng Lake is not only a beautiful natural landscape, but also a place with profound cultural significance that attracts tourists every year. This trend may reflect the occurrence of small microplastics in the areas where tourist attractions and hotels exist.

### 3.3 Size of sub-20 μm microplastics

**Figure 4:**
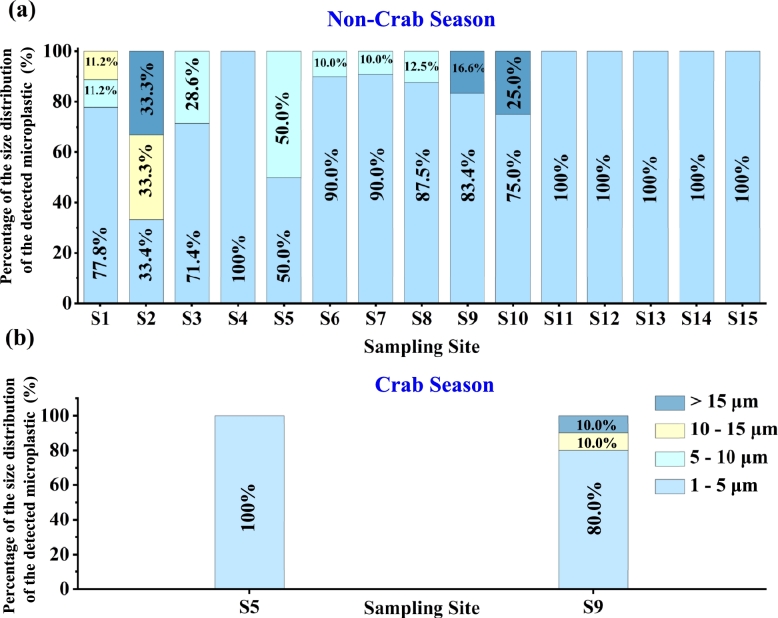
**Microplastics size distribution versus sampling site for:** (a) Non-crab season and (b) crab season. The category of 1 - 5 μm is the most dominant size for each size sampling. Note that the graphs (a) and (b) did not include copolymerized plastics. The size of microplastic particles with multiple polymer compositions is: Polystyrene and Polyethylene (PS/PE): 4.6 (short size)/6.9 μm (long side); and Polystyrene and Polymethyl methacrylate (PS/PMMA): 4.9 (short size)/25.4 μm (long size).

The size of the microplastics detected in all the water samples is divided into four categories as shown in Fig. 4. Plastics in the categories 1 - 5 μm were found at every sampling site during both seasons. Microplastic particles of 5 - 10 μm were found at six sampling sites (S1, S3, S5 (non-crab season)-S8). Plastics in the category 10 - 15 μm were detected in three sampling sites (S1, S2, S9 (crab-season)), and in the category larger than 15 μm (4 plastics) were detected in three sampling sites (S2, S9, S10). Since a 125 μm sieve has been used to remove the large microplastics and macroplastics; it is expected that most plastics will be within the range of small microplastics [62]. Note that the average size of the plastic particles with multiple polymer compositions is 4.6/6.9 μm (PS/PE-detected at station S7) and 4.9/25.4 μm (PS/PMMA-detected at station S2).

Among the three zones of the Yangcheng Lake, the stations in the middle zone, along with station S4, show the highest proportion of detected microplastics smaller than 5 μm (100%), while station S2 has the lowest proportion (33.4%). Additionally, the level of microplastics within this size range decreases at station S9 during the crab season (80%), whereas it increases at station S5 (100%). The observed variation in percentage of the size distribution at stations S9 and S5 during the crab season may be related to seasonal changes in water dynamics. A possible explanation is that at station S9, the decrease in the percentage of the microplastic size during the crab season (from 83.5% to 80.0%) may be due to increased water movement from fishing activities dispersing these particles away from the collected area or causing them to settle more rapidly. On the other hand, the increase in the percentage of microplastic size at station S5 (from 50% to 100%) may be to similar water dynamics stirring up and redistributing microplastics, leading to a higher percentage detected in the area. However, the small volume of the freshwater examined in this work is limited for a comprehensive quantification study and may not provide enough data to accurately determine the size distribution of small microplastics. Therefore, further experiments are needed to better understand the factors affecting the distribution of microplastic sizes in Yangcheng Lake and will be the focus of future study.

### 3.4 Shape of sub-20 μm microplastics

**Figure 5:**
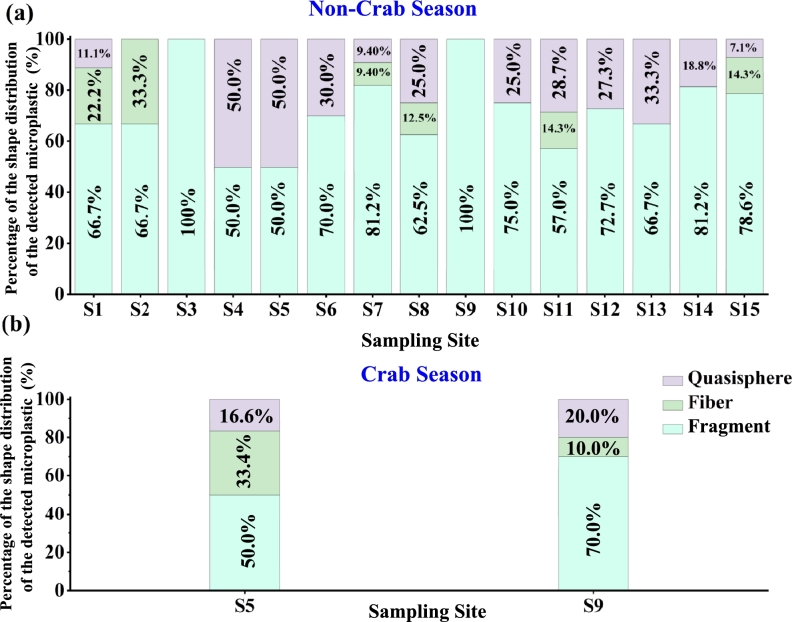
**Distribution of microplastics' shape as a function of sampling site for:** (a) Non-crab season and (b) Crab season. The most dominant shape of the detected microplastics in this study is fragments.

The shape of microplastics detected in water samples was classified into three categories based on images collected during the evaluation process: quasispheres, fragments, and fibers (SI-S7). Quasisphere microplastics are round particles with a pellet shape [63]. Fragment microplastics were isolated or incomplete portions of huge plastic debris, usually with irregular shape [63]. Fiber microplastics were defined as microplastics with a slender and highly elongated look [63]. The distribution of microplastic particles of different shapes is shown in Fig. 5. Quasisphere plastics were detected in 12 sample sites, ranging from 7.1% - 50.0%. Fragments are found in every sampling site and are the most abundant among the three shapes with a percentage between 50.0% (S4) - 100.0% (S3 and S9 during non-crab season) of all detected plastics. Fibers were present in eight sample sites and accounted for 9.4% - 33.3% of all detected plastics during non-crab season. Note that the concentration of fibers is relatively high at sampling sites S5 (33.4% crab season and 0% non-crab season) and S2 (33.3%) of the total plastics that were detected in these two stations. This might be due to the large use of crab nets, ropes, and fishing lines in crab culturing areas around S5, and disposable packaging, knots, and other worship supplies largely used in station S2, making these two sampling sites more exposed to fiber production. The source of fibers detected in station S11 could be due to crab culturing activities. During crab seasons, the concentration of the fiber increased in station S5 and the concentration of quasisphere and fiber plastics increased in station S9. Both of these might be due to excessive fishing tools due to frequent crabbing. The results also show that fragments are the most abundant shapes in Yangcheng Lake freshwater samples. Fragments mainly come from the fragmentation of large plastic items under the effect of mechanical forces and photochemical processes. Similar observations have been reported in various freshwater [39], [64], [65], [66] and marine [67], [68], [69] ecosystems globally, indicating the pervasive nature of plastic fragmentation. In this study, the majority of small quasisphere microplastics range from 1.1 μm (PS) to 8.5 μm (PVC) and small fiber microplastics range from 1.1 μm (PS) to 11.9 μm (PE) (SI S8-Table S7). This size distribution may highlight the variability in microplastic particle sizes, influenced by the type of polymer and the processes leading to their fragmentation.

Compared with data from previous studies (Table 3), this work found that the most abundant microplastic shapes were fragments and quasispheres, rather than fibers. This deviation can be attributed to several factors related to methodology, sample handling, and environmental conditions. Methods involving filtering or sieving may preferentially capture shapes like fibers while potentially missing smaller fragments. The approach used in this study, which avoids extensive sample preparation, may be more effective in capturing a broader range of microplastic shapes. Additionally, traditional analytical techniques, such as FTIR and standard Raman spectroscopy, have limitations in size. On the other hand, the optical tweezers combined with micro-Raman spectroscopy used in this study provide high precision in trapping and analyzing smaller particles, enabling a more detailed characterization of microplastic sizes and/or shapes. Furthermore, the unique environmental conditions of Yangcheng Lake, including its specific degradation processes, could contribute to a high proportion of small microplastics fragments and quasispheres compared to other locations. Despite these differences, the general sources of microplastic contamination remain similar across various studies, particularly in urban and industrial settings, leading to consistent polymer compositions.

**Table 3:** **Comparison of microplastic characteristics in the surface freshwaters detected in this study with other lakes**. Note: PS : polystyrene, PE : polyethylene, PVC : polyvinyl chloride, PP : polypropylene, PET : polyethylene terephthalate.

Location	Identification	Dominant shape	Size range	Main polymers	References
Dianchi Lake	*μ*-Raman	Fiber	0.100 - 5.000 mm	PE and PET	[70]
Tibet Lake	*μ*-FTIR	Fiber and film	0.300 - 1.000 mm	PP and PET	[71]
Taihu Lake	*μ*-FTIR	Fiber	0.100 - 1.000 mm	Cellophane	[39]
Poyang Lake	*μ*-Raman	Fiber and film	0.100 - 0.500 mm	PP and PE	[40]
Dongting Lake	*μ*-Raman	Fiber	0.050 - 5.000 mm	PP and PE	[41]
Mongolia Lake	*μ*-Raman	Fiber and fragment	0.050 - 0.200 mm	PP and PE	[38]
Hong Lake	*μ*-Raman	Fiber	0.050 - 5.000 mm	PP and PE	[41]
Wuliangsuhai Lake	FTIR	Fiber	0.075 - 0.500 mm	PS and PE	[51]
Urban Lake of Wuhan	FTIR	Fiber	0.500 - 2.000 mm	PE and PP	[61]
Qinghai Lake	*μ*-Raman	Fiber and sheet	0.112 - 0.500 mm	PE and PP	[33]
Yangchen Lake	OT*μ*-Raman	Fragment and quasisphere	0.001 - 0.009 mm	PS and PE	this study

As there is no uniform standard for recording microplastic occurrence in freshwater, more studies should focus on the sub-20 μm range so the comparison of the microplastic occurrence in different regions can be more accurate. In this study, an optical tweezer micro-Raman Spectroscopy (OT*μ*-RS) technique has been employed to detect and characterize sub-20 μm microparticles in freshwater ecosystems. However, this technique can only be qualitative and not quantitative. To use this method for quantifying the concentration of particles per liter, additional modifications would be necessary, which are beyond the scope of this study. For instance, a larger proportion of the total sample volume would need to be analyzed, potentially using a high-throughput system to ensure more representative sampling and accurate quantification or using automatic particle detection to provide reliable plastic abundance distributions. In addition, micro-Raman spectroscopy with plasmonic optical tweezers may help detect nanoplastic pollution in freshwater ecosystems. Plasmonic optical tweezers [72], [73], [74], using the strong electromagnetic fields generated by metallic nanostructures, can trap and manipulate nanoparticles with high precision. The combination of these two approaches will enable us to not only detect but also study the behavior and impact of nanoplastics in freshwater ecosystems, and this will be the focus of future work.

Closing, microplastic ecological risks are influenced by several factors, including the size, abundance, and type of polymer [37]. The quasisphere microplastics identified in this study ranged from 1.1 μm to 8.5 μm, which are similar in size to zooplankton and may pose potential risks to zooplanktivorous predators [75]. However, due to the lack of the abundance of small microplastics per liter calculations, we cannot reach a definitive conclusion about their ecological impact. Furthermore, the chemical composition of microplastics, along with any adhered pollutants, can lead to significant chemical and biological impacts, affecting toxicity to organisms [37]. In our study, we identified five types of polymers, with PVC (9 particles) classified as highly hazardous polymer type [49]. However, since PVC constituted only 9 out of 136 detected microplastics within the sample volume analyzed, we cannot draw definitive conclusions about ecological risks. In order to better assess microplastics' potential ecological impacts in freshwater system of Yangcheng Lake, further studies with comprehensive abundance calculations may be required.

## 4 Conclusions

The above results provide valuable insight into the analysis and identification of microplastics in Yangcheng Lake, highlighting a significant contamination issue with sub-20 μm microplastics. The study found that approximately 26.5% of detected particles were small microplastics, indicating widespread presence throughout the lake. Sub-20 μm microplastics were consistently found across all examined locations, with their abundance varying per sampled volume and being more frequent in regions with high human activity. A closer examination revealed that microplastics in the size range of 1.1 - 8.5 μm were mostly found near restaurant areas and crab culturing areas. Most of these microplastics were fragmented pieces, with polystyrene (PS) and polyethylene (PE) identified as the dominant polymer types. Additionally, an increase in microplastic contamination during crab season is observed, attributed to the increased use of plastic materials in crab farming. As well the risks that sub-20 μm microplastics pose to crabs and their natural food sources, especially invertebrates, and the possible link to human health, need to be better understood. To mitigate these risks, strategies such as proper waste management, plastic recycling, and penalties for illegal dumping in areas close to water resources should be encouraged and implemented in the communities. These measures could reduce the land-based microplastics that end up in freshwater bodies, thus protecting the aquatic ecosystem and potentially improving public health outcomes.

## CRediT authorship contribution statement

**Zhenyu Xu:** Writing – review & editing, Writing – original draft, Methodology, Investigation, Formal analysis, Data curation. **Natalie Earnhardt:** Writing – review & editing, Validation, Methodology, Data curation. **Domna G. Kotsifaki:** Writing – review & editing, Supervision, Resources, Methodology, Investigation, Funding acquisition, Conceptualization.

## Declaration of Competing Interest

The authors declare that they have no known competing financial interests or personal relationships that could have appeared to influence the work reported in this paper.

## Data Availability

Data will be made available on request.

## Appendix A Supplementary material

MMC Supporting Information “Detection of small microplastics in the surface freshwater samples of Yangcheng Lake, China”: S1 Sampling sites (Table S1); S2 Raman modes of plastic particles detected in this study (Tables S2, S3, S4, S5, S6); S3 Raman spectra of trapped microplastics embedded in organic matter (Fig. S1); S4 Calculations on an average increase of the number of microplastic particles per station per season (Fig. S2); S5 Calculations of the small particle occurrence; S6 Satellite images of villages around Yangcheng Lake (Fig. S3); S7 Optical microscope images of different shapes of microplastics (Fig. S4); S8 Polymer sizes as found in microplastics in Yangcheng Lake freshwater samples (Table S7). MMC
