# Supplementary material for: Detection of small microplastics in the surface freshwater samples of Yangcheng Lake, China
Source: Heliyon. 2024 Oct 28;10(21):e39779. doi: 10.1016/j.heliyon.2024.e39779 (PMC11565016; doi:10.1016/j.heliyon.2024.e39779)
Supplement: MMC — Supporting Information “Detection of small microplastics in the surface freshwater samples of Yangcheng Lake, China”: S1 Sampling sites (Table S1); S2 Raman modes of plastic particles detected in this study (Tables S2, S3, S4, S5, S6); S3 Raman spectra of trapped microplastics embedded in organic matter (Fig. S1); S4 Calculations on an average increase of the number of microplastic particles per station per season (Fig. S2); S5 Calculations of the small particle occurrence; S6 Satellite images of villages around Yangcheng Lake (Fig. S3); S7 Optical microscope images of different shapes of microplastics (Fig. S4); S8 Polymer sizes as found in microplastics in Yangcheng Lake freshwater samples (Table S7). [file mmc1.docx]

**Supporting Information**

**Detection of small microplastics in the surface**

**freshwater samples of Yangcheng Lake,**

**China**

Zhenyu Xu, Natalie Earnhardt, and Domna G. Kotsifaki*

*Photonics Lab, Division of Natural and Applied Sciences, Duke Kunshan University, 8 Duke Ave, Kunshan, Jiangsu Province, 215316, China*

*E-mail:* [*domna.kotsifaki@dukekunshan.edu.cn*](mailto:domna.kotsifaki@dukekunshan.edu.cn)*;* [*dk310@duke.edu*](mailto:dk310@duke.edu)

**S1 Sampling Sites**

Table S1: Location of the sampling sites in the Yangcheng Lake, China

| Area name | Station names |
| --- | --- |
| Huayi film center | S1 |
| Chonyuan temple | S2 |
| Hotel | S3 |
| Organic Farm | S4 |
| Crab restaurants | S5 |
| Yangcheng East Lake Wetland Park | S6 |
| Waihetou village | S7 |
| Maozhuang village | S8 |
| Zhongxiang village | S9 |
| Yulan Park | S10 |
| Hotel | S11 |
| Nan village | S12 |
| Qianye camping site | S13 |
| Yiyuan dock | S14 |
| Shangxi dock | S15 |

**S2 Raman modes of plastic particles detected in this study**

Table S2: Raman modes of polystyrene (PS)

| **PS: Wavenumber (cm^-1^)** | **Vibration** |
| --- | --- |
| 622 | Aromatic *C = C* in plane ring deformation |
| 793 | Aromatic *C = C* out of plane deformation |
| 998 | Aromatic ring breathing mode |
| 1026 | Aromatic *C-H* in plane deformation |
| 1152 | Aromatic *C-H* in plane deformation |
| 1183 | Aromatic ring stretching |
| 1437 | *CH_2_* deformation |
| 1588 | Aromatic *C=C* stretching |

Table S3: Raman modes of polyethylene (PE)

| **PE: Wavenumber (cm^-1^)** | **Vibration** |
| --- | --- |
| 1058 | *CC* symmetric stretching |
| 1123 | *CC* anti-sym stretching |
| 1286 | *CH_2_* twisting vibration |
| 1408 | *CH_2_* bending |
| 1429 | *CH_2_* symmetric deformation |
| 1450 | *CH_2_* scissor vibration |

Table S4: Raman modes of polyvinyl chloride (PVC)

| **PVC: Wavenumber (cm^-1^)** | **Vibration** |
| --- | --- |
| 610 | Crystalline *C-Cl* stretching |
| 637 | Crystalline *C-Cl* stretching |
| 694 | Non crystalline *C-Cl* stretching |
| 1302 | *CH_2_* twisting |
| 1325 | *CH_2_* twisting |
| 1422 | *CH_2_* bending |
| 1434 | *CH_2_* symmetric deformation |
| 1724 | Ester *C=O* stretching |

Table S5: Raman modes of polyamide-6 (NY6)

| **NY6: Wavenumber (cm^-1^)** | **Vibration** |
| --- | --- |
| 950 | *C-CO* stretching |
| 1052 | *CC* asymmetric stretching |
| 1124 | *CC* anti-sym stretching |
| 1290 | *CH_2_* twisting and *CN* stretching and *NH* bending |
| 1431 | *CH_2_* symmetric deformation |
| 1465 | *CH_2_* scissor vibration |
| 1633 | *C=O* stretching in amide I |

Table S6: Raman modes of polymethylmetaacrylate (PMMA)

| **PMMA: Wavenumber (cm^-1^)** | **Vibration** |
| --- | --- |
| 601 | *C-C-O* stretching |
| 810 | *C-O-C* symmetric stretching |
| 961-985 | *O-CH_3_* rocking |
| 1441 | *C-H* bending |
| 1712 | *C=O* stretching |

**S3 Raman spectra of trapped microplastics embedded in organic matter**

**Figure S1: Raman spectra of microplastic particles embedded in organic matter.** Up (dark blue): Raman spectra of a PE particle detected in sampling station S5. Below (light blue): Raman spectra of a PE particle embedded in organic matter detected in sampling station S6, showing fluctuations and additional peaks.

**S4 Calculations on an average increase of the number of microplastic particles per station per season**

**Figure S2:** **Number of microplastic particles detected in surface Yangcheng Lake freshwater samples from station S5 (crab restaurant) and station S9 (Zhongxiang village) during non-crab season (Ⅰ) and crab season (Ⅱ).** The increase in the number of sub-20 μm plastics in S5 is 4 plastics per station per season. Therefore the increase of sub-20 μm plastics is 4 plastics per station per season within the sample volume analyzed.

**S5 Calculations of the small particle occurrence**

The occurrence of small microplastics in our study represents the proportion of microplastics among all the particles analyzed within the selected volume.

For example, the occurrence of small microparticles in station S1 is calculated as:

$$\left( \frac{Number of small plastics detected in station S1}{Number of all detected particles in station S1} \right)100\%=\left( \frac{9}{9+21} \right)100\%=\left( \frac{9}{30} \right)100\%=30\%$$

**S6 Satellite images of villages around Yangcheng Lake**


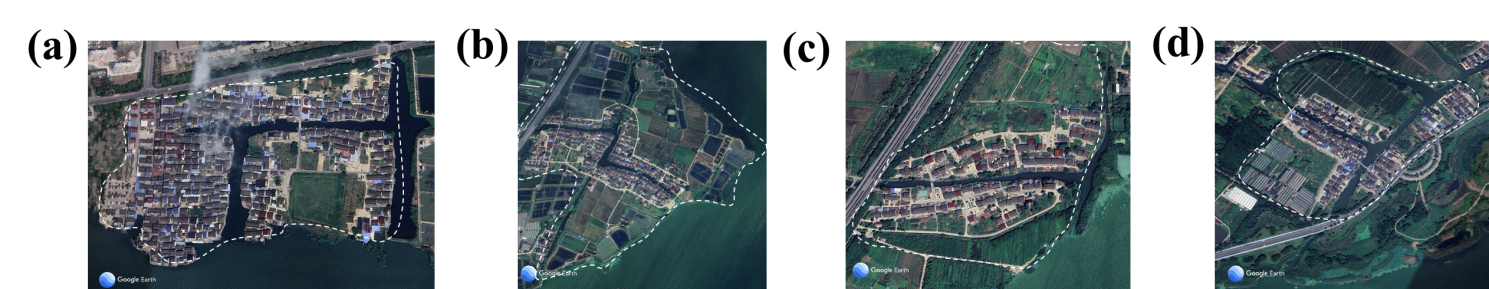


**Figure S3:** **Satellite images of villages from Google Earth.** (a) S7: Waihetou village (31^o^29’17.18’’N, 120^o^45’01.95’’E). Farmland is surrounded by high-density residential areas. (b) S8: Maozhuang village (31^o^26’50.36’’ N, 120^o^43’19.84’’ E). Large area of farm lands separated from the river branch by residential houses. (c) S9: Zhongxiang village (31^o^24/38.65’’N, 120^o^41’24.94’’E). There is a low density of houses and a small area of farmland. (d) S12: Nan village (31^o^25’55.62’’N, 120^o^44’53.59’’E). Plastic greenhouses are used on the farm lands.

**S7 Optical microscope images of different shapes of microplastics**


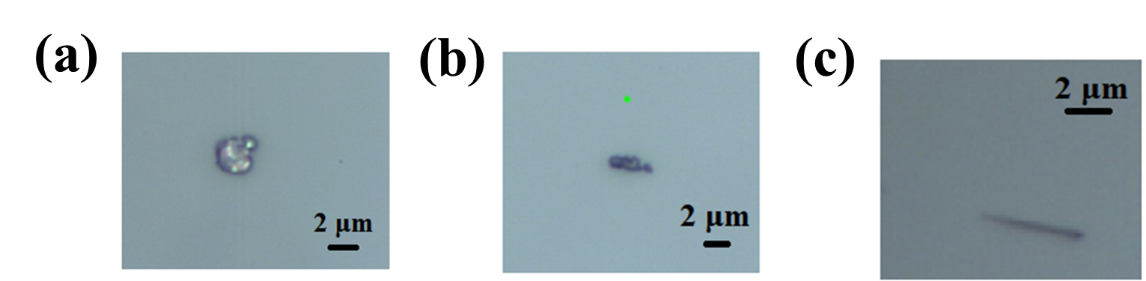


**Figure S4**: **Optical images of optical trapped microplastics**. (a) A quasisphere polystyrene plastic with an average size of 3.6 μm. (b) A fragment of polyethylene plastic of 3.4 μm average size. (c) A polyvinyl chloride plastic with a long diameter of 5.7 μm.

**S8 Polymer sizes as found in microplastics in Yangcheng Lake freshwater samples.**

Table S7: Size range includes the diameter of quasisphere particles and the length of fiber particles. For fragments with irregular shapes, the magnitude of their long side size and short side size are included.

| **Polymer Type** | **Range of Quasisphere**  **Diameter (*μm*)** | **Range of Fiber**  **Length (*μm*)** | **Range of Fragment**  **Long side (*μm*)** | **Range of Fragment**  **Short side (*μm*)** |
| --- | --- | --- | --- | --- |
| PS | 1.1 – 3.1 | 1.1 – 5.9 | 2.4 – 10.5 | 1.3 – 3.9 |
| PE | 1.2 – 1.6 | 3.3 – 11.9 | 2.9 – 22.6 | 1.2 – 13.3 |
| PVC | 1.9 – 8.5 |  | 4.3 – 48.7 | 3.3 – 25.9 |
| NY6 | 1.1 – 2.2 | 1.2 – 3.6 | 7.7 | 2.6 |
| PMMA | 1.3 – 3.4 |  | 4.9 – 5.8 | 3.3 – 3.9 |

**REFERENCES**

1. R. Gilibert, G. Balakrishnan, Q. Deshoules, M. Tardivel, A. Magazzú, M. G. Donato, O. M. Maragò, M. Lamy de La Chapelle, F. Colas, F. Lagarde, et al., Raman tweezers for small microplastics and nanoplastics identification in seawater, Environmental Science & Technology 51 (15) (2019) 9003-9013.
2. C. Ripken, D. G. Kotsifaki, S. Nic Chormaic, Analysis of small microplastics in coastal surface water samples of the subtropical island of Okinawa, Japan, Science of the Total Environment 760 (2021) 143927.
